# Hyperleptinemia, Adiposity, and Risk of Metabolic Syndrome in Older Adults

**DOI:** 10.1155/2013/327079

**Published:** 2013-12-26

**Authors:** Suruchi Mishra, Tamara B. Harris, Trisha Hue, Iva Miljkovic, Suzanne Satterfield, Nathalie de Rekeneire, Mira Mehta, Nadine R. Sahyoun

**Affiliations:** ^1^Department of Nutrition and Food Science, University of Maryland, College Park, MD 20742, USA; ^2^National Institute on Aging, NIH, Bethesda, MD 20892, USA; ^3^University of California, San Francisco, CA 94107, USA; ^4^Department of Epidemiology, University of Pittsburg, Pittsburgh, PA 15213, USA; ^5^Memphis Field Center, University of Tennessee, Memphis, TN 38163, USA; ^6^Center on Disability and Disabling Disorders, New Haven, CT 06520, USA

## Abstract

*Background*. Abdominal adiposity and serum leptin increase with age as does risk of metabolic syndrome. This study investigates the prospective association between leptin and metabolic syndrome risk in relation to adiposity and cytokines. *Methods*. The Health, Aging, and Body Composition study is a prospective cohort of older adults aged 70 to 79 years. Baseline measurements included leptin, cytokines, BMI, total percent fat, and visceral and subcutaneous fat. Multivariate logistic regression was used to determine the association between leptin and metabolic syndrome (defined per NCEP ATP III) incidence after 6 years of follow-up among 1,120 men and women. *Results*. Leptin predicted metabolic syndrome in men (*P* for trend = 0.0002) and women (*P* for trend = 0.0001). In women, risk of metabolic syndrome increased with higher levels of leptin (compared with quintile 1, quintile 2 RR = 3.29, CI = 1.36, 7.95; quintile 3 RR = 3.25, CI = 1.33, 7.93; quintile 4 RR = 5.21, CI = 2.16, 12.56; and quintile 5 RR = 7.97, CI = 3.30, 19.24) after adjusting for potential confounders. Leptin remained independently associated with metabolic syndrome risk after additional adjustment for adiposity, cytokines, and CRP. Among men, this association was no longer significant after controlling for adiposity. *Conclusion*. Among older women, elevated concentrations of leptin may increase the risk of metabolic syndrome independent of adiposity and cytokines.

## 1. Introduction

Leptin is an adipocyte-derived hormone that influences appetite [1] and reflects amount of energy stored in adipose tissues [2, 3]. The association between plasma leptin and fat mass is disrupted with aging [4]. Aging is accompanied by an increase in serum leptin levels [2], proinflammatory cytokines, such as TNF-*α* [5, 6], and prevalence of metabolic syndrome [7].

Leptin also plays a critical role in the inflammatory response [8] and has been associated with insulin resistance [9] and metabolic syndrome [7]. Leptin directly inhibits insulin secretion from pancreatic *β*-cells [10] and elevated serum leptin levels are associated with fasting insulin, insulin resistance (HOMA-IR), and total cholesterol [11–15], the core metabolic disturbances of the metabolic syndrome. Although the proinflammatory cytokines are associated with serum leptin and have been implicated in the development of metabolic syndrome, the prospective association of serum leptin with metabolic syndrome has not been explored independently of proinflammatory cytokines and body fat depots among older adults.

The primary objective of this study is to examine the association between serum leptin and the development of metabolic syndrome over a 6-year follow-up in a cohort of older adults and to examine whether such an association is independent of markers of inflammation and body fat depots.

## 2. Subjects and Methods

### 2.1. Study Design

The Health, Aging, and Body Composition (Health ABC) study is a longitudinal cohort study of 3,075 community dwelling, well-functioning white and black men and women aged 70 to 79 years at study commencement. Participants were recruited for the study from a random sample of white residents receiving Medicare benefits and all age-eligible black residents of Pittsburgh, Pennsylvania, and Memphis, Tennessee. Subjects were considered eligible to participate in the study if they reported no difficulty walking a quarter of a mile, climbing 10 steps without resting, or performing mobility-related activities of daily living, were free of life-threatening illness, and planned to remain in the area for at least 3 years. Participants gave written informed consent, and protocols were approved by the Institutional Review Boards at the two study sites. A home interview was administered to collect information on demographic and socioeconomic factors and health behaviors. A clinic visit at baseline was conducted for examination of biological variables, body composition, weight-related health conditions, and physical function between April 1997 and June 1998. Thereafter, follow-up clinical examinations have been conducted annually for six years, then every other year until 10 years of follow-up, and interviews semiannually.

### 2.2. Subjects

In the present study, data from baseline and year 6 of the Health ABC study were used. The exclusion criteria were having prevalent metabolic syndrome at baseline (*n* = 773), having an energy intake of less than 800 kcal/day or more than 4,000 kcal/day among men (*n* = 88), and having an energy intake of less than 500 kcal/day or more than 3,500 kcal/day (*n* = 64) among women. Participants who died prior to year 6 (*n* = 790) or had missing or implausible values for the metabolic syndrome at year 6 (*n* = 240) or missing control variables such as serum leptin (55) and plasminogen activator inhibitor 1 (65) were excluded. There was no significant difference in leptin, cytokines, and fat measures between those who had data for metabolic syndrome at year 6 and those who were alive but did not have data for the outcome variable. The total sample size for the analysis was 1120 (552 males and 568 females). One hundred thirteen participants were missing data for total percent fat, 119 for abdominal visceral fat, 209 for abdominal subcutaneous fat, 203 for TNF-*α*, 38 for C-reactive protein, and 162 for IL-6. The number of participants missing information for these variables did not differ by the outcome variable (metabolic syndrome at year 6).

### 2.3. Biochemical Variables

Participants underwent venipuncture at baseline visit after an overnight fast, and serum samples were frozen at −70°C. Serum concentrations of leptin were measured in duplicate by means of sensitive human leptin Radioimmunoassay Kit (RIA Kit) (product number SHL-81 K) from Linco Research Inc. (St. Charles, MO) [16]. The assay is highly specific for human leptin and shows minimal reactivity with mouse or rat leptin. Plasma glucose levels were measured using an automated glucose oxidase reaction (YSI 2300 Glucose Analyzer; Yellow Springs Instruments, Yellow Springs, OH). Triglyceride (TG) and high-density lipoprotein cholesterol (HDL-C) levels were measured using a chemical analyzer (Vitros 950; Johnson & Johnson, Raritan, NJ). Both interleukin-6 (IL-6) and tumor necrosis factor-*α* (TNF-*α*) levels were measured in duplicate using an ultrasensitive enzyme-linked immunosorbent assay (R&D Systems, Minneapolis, MN). The limit of detection was 0.10 pg/mL for IL-6 and 0.18 pg/mL for TNF-*α*. The level of plasminogen activator inhibitor-1 (PAI-1) was measured in citrated plasma samples using a 2-site enzyme-linked immunosorbent assay. Serum levels of C-reactive protein (CRP) were also measured in duplicate by enzyme-linked immunosorbent assay based on purified protein and polyclonal anti-CRP antibodies (Calbiochem, San Diego, CA).

### 2.4. Anthropometric and Clinical Variables

Weight in kilograms was measured using a standard balance beam scale. Height in centimeters was measured using a Harpenden stadiometer (Holtain Ltd., Crosswell, UK) with a maximum of four measurements taken and the average of the two close measurements was used. BMI (weight (kg)/height (m^2^)) was calculated. At baseline, axial computerized tomography (CT) scanning of abdomen was performed. Abdominal visceral and subcutaneous fat were quantified from scans, performed on a General Electric 9800 Advantage (Milwaukee, WI) in Pittsburgh and a Siemens Somatom Plus (Iselin, NJ) and Picker PQ2000S (Cleveland, OH) in Memphis. Data from CT scans were analyzed at the University of Colorado Health Sciences Center according to a standardized protocol [17, 18]. Dual energy X-ray absorptiometry (DXA) (Hologic QDR 4500A, software version 8.21, Hologic, Waltham, MA) was used to assess the total fat mass and total percentage of body fat was calculated. Systolic and diastolic blood pressures were measured by manual sphygmomanometer in a seated position.

### 2.5. Demographic and Life Style Variables

A standardized questionnaire was administered at baseline to collect information on sociodemographic variables including age, gender, self-identified race, years of education, and lifestyle variables including smoking status and average alcohol consumption (0, 1-2, or >2 drinks/day) during the past year. The pack-years over a lifetime of cigarette smoking were calculated. Level of physical activity was obtained using a standardized questionnaire specifically designed for the Health ABC study [19].

### 2.6. Outcome Variable

In Health ABC, metabolic syndrome at year 6 was defined according to the National Cholesterol Education Program, Adult Treatment Panel III (NCEP ATP III), as the presence of 3 or more of the 5 risk components as follows: (1) waist circumference >102 cm for men and >88 cm for women; (2) TG ≥ 150 mg/dL; (3) HDL-C <40 mg/dL for men and <50 mg/dL for women; (4) blood pressure (BP) ≥130/85 mmHg or currently on antihypertensive medicine(s); (5) fasting glucose ≥110 mg/dL or currently using antidiabetic (insulin or oral agents) medication.

### 2.7. Statistical Analysis

Serum leptin values and body fat vary by gender; therefore, the analysis was performed separately by gender [20]. Baseline characteristics of men and women were examined by quintiles of serum leptin levels. For continuous variables, least squares means were computed with Dunnett's test option, and, for categorical variables, the chi-square test was used to compare the means of quintiles 2 through 5 to those of quintile 1. All adipokines were log-transformed because they were not normally distributed. Pearson's correlation coefficients of leptin with anthropometric parameters and cytokines were calculated. There were no significant interactions of serum leptin with race, proinflammatory cytokines, and CRP or adiposity measures. Multivariate logistic regression modeling was used to determine the association between baseline serum leptin and risk of developing metabolic syndrome at year 6 of follow-up. Quintiles 2 through 5 of serum leptin were compared to quintile 1 in 3 different models. The first model was adjusted for baseline descriptive variables (age, race, study site, education, smoking status, alcohol use, physical activity, calorie intake, hormonal replacement therapy, and number of hours fasted when blood was drawn). The second model was adjusted for variables in model 1 and body fat (abdominal fat, total fat percent, and BMI). The association between leptin and metabolic syndrome remained the same when BMI was replaced in the model with total percent fat, visceral fat, or subcutaneous fat so they were not included in the subsequent analysis. The third model was adjusted for variables in model 2 and markers of systemic inflammation (CRP, TNF-*α*, IL-6, and PAI-1). TNF-alpha and IL-6 were not significant and did not affect the association between main predictor and the outcome, so they were not included in the final analysis. Log-transformed leptin was used in regression model for testing a linear trend. Further, the effect of body adiposity on the association of serum leptin with metabolic syndrome was stratified by two BMI categories (BMI ≤ 25 and BMI > 25) using multivariate logistic regression analysis adjusted for potential confounders. There were no interactions between leptin quintiles and BMI categories when metabolic syndrome was the outcome. Statistical significance was set at *P* ≤ 0.05, and analysis was performed using SAS (version 9.1; SAS Institute Inc., Cary, NC).

## 3. Results

Mean leptin concentrations differed by gender, men 7.1 ng/mL (±5.8 ng/mL) and women 18.7 ng/mL (±13.3 ng/mL) (*P* < 0.0001). Baseline characteristics indicate that men and women in quintile 1 had significantly lower BMI, percent body fat, and visceral and abdominal subcutaneous fat as compared to all other quintiles (Tables 1 and 2). Men in the lowest quintile of serum leptin also had significantly lower abdominal circumference than those in quintiles 3, 4, and 5 and lower fasting glucose, CRP, and PAI-1 than those in quintiles 4 and 5. Additionally, men in quintile 1 had significantly lower IL-6 than men in quintile 4 and lower TNF-*α* than men in quintile 5 (Table 1).

The percentage of white women as well as the level of education among women in quintile 1 was significantly higher than quintiles 4 and 5. Fasting glucose, CRP, and PAI-1 were significantly lower among women in the lowest quintile than among those in the two highest quintiles. IL-6 and TNF-*α* were also significantly lower among women in leptin quintile 1 than those in quintiles 4 and 5, respectively (Table 2).

Among men, measures of adiposity (*P* < 0.0001, *r* ranged from 0.52 to 0.71), cytokines (*P* < 0.001, *r* ranged from 0.08 to 0.34), and CRP (*P* < 0.0001, *r* = 0.11) were significantly correlated with serum leptin and used in further analyses. Among women, measures of adiposity (*P* < 0.0001, *r* ranged from 0.45 to 0.68), cytokines (*P* < 0.0001, *r* ranged from 0.12 to 0.26), and CRP (*P* < 0.0001, *r* = 0.33) were significantly correlated with serum leptin and used in further analyses (not tabulated).

For both men and women, the incidence of metabolic syndrome was significantly associated with leptin as a continuous variable (*P* for trend 0.0076 and 0.0003, resp.) (Tables 3 and 4). In multivariate analysis, men in quintile 5 of serum leptin were at higher odds of developing metabolic syndrome at year 6 as compared to those in quintile 1 after adjusting for potential confounders. However, the association was no longer significant when the analysis was additionally adjusted for BMI in model 2 and cytokines and CRP in model 3 (Table 3).

Women in quintiles 2, 3, 4, and 5 of leptin were at significantly higher risk of developing metabolic syndrome at year 6 as compared to those in quintile 1 after adjusting for potential confounders. When the analysis was further adjusted for adiposity measures in model 2 and cytokines and CRP in model 3 there was some attenuation in the association between leptin and the risk of metabolic syndrome, but the results remained significant. The risk of metabolic syndrome and serum leptin was linearly associated in models 1 (*P* = 0.0001), 2 (*P* = 0.0024), and 3 (*P* = 0.0098) (Table 4).

Since with age body fat is expected to increase in a period of 6 years, therefore, we tested the association between baseline leptin and incidence of metabolic syndrome controlling for adiposity at baseline and also at the 6-year period. In women, log leptin remained significantly associated with the risk of metabolic syndrome at year 6 after adjustment for potential confounders and adiposity measures (BMI, abdominal visceral fat, and total percent fat) at year 6 (OR 1.75; CI 1.004 to 3.032; *P* = 0.04). However, the association was still not significant among men (OR 0.63; CI 0.28 to 1.37; *P* = 0.88).

Among men, the leptin was not associated with the risk of developing metabolic syndrome at year 6 in both BMI categories (BMI ≤ 25 and BMI > 25). Among women, leptin was significantly associated with the risk of metabolic syndrome at year 6 in both BMI groups after adjusting for potential confounders (Table 5).

## 4. Discussion

In the present study, higher levels of serum leptin concentrations were positively associated with the risk of developing metabolic syndrome in older women independent of potential confounders, measures of body fat distribution, proinflammatory cytokines, and CRP.

Few longitudinal and several cross-sectional studies examined the association between serum leptin and metabolic disorders. In middle-aged adults leptin was a predictor of the metabolic syndrome, insulin resistance, and hyperinsulinemia [11] and positively associated with metabolic syndrome independent of measures of body fat [21]. The gender-specific differences in the association of leptin with the risk of metabolic syndrome were not examined in these longitudinal studies. Additionally, serum leptin levels have been linked to individual components of the metabolic syndrome in cross-sectional studies [12, 22–26].

Data from cross-sectional studies suggest a gender disparity in the association between serum leptin levels and metabolic disorders [7, 27, 28]. The association between leptin and insulin resistance was shown to be independent of total fat mass and BMI among women in another cross-sectional study [28]. On the other hand, the association of serum leptin with lipid parameters was mediated by BMI among middle-aged men in a cross-sectional study [7]. Studies have consistently shown that women have higher serum leptin concentrations independent of total body fat mass or percent fat [20, 29]. Compared to older men, higher serum leptin concentrations in older women were not fully explained by visceral and subcutaneous adipose tissues [2, 30]. It was proposed that elevated levels of serum leptin can lead to the development of leptin resistance [31] suggesting that women may be more leptin resistant compared to men. Thus, the gender difference in the association of serum leptin with metabolic syndrome in our study may be explained by relatively greater leptin insensitivity in women. The independent association between serum leptin and metabolic syndrome among older women also suggests that the etiology of metabolic syndrome among men and women may be somewhat different and require separate approaches for its prevention.

In this study, serum leptin remained an independent predictor of metabolic syndrome among women with BMI below 25 and those above it. Previous studies on the role of adiposity on the association of serum leptin and metabolic disorders have shown inconsistent results. The inverse association between serum leptin and increased risk of diabetes was independent of obesity in the Atherosclerosis Risk in Communities (ARIC) [32], whereas it was mediated by adiposity and insulin insensitivity in a study among Japanese men and women [24]. Similarly, association between serum leptin, insulin resistance, and metabolic syndrome was mediated through central obesity in a cross-sectional study on Iranian population [33]. A positive association between elevated leptin and history of myocardial infarction among men and women and stroke among women was independent of obesity in study on National Health and Nutrition Examination Survey (NHANES) data [34].

Although serum leptin has been linked to proinflammatory cytokines [5, 6] and enhanced productions of proinflammatory cytokines have been implicated in the development of metabolic disorders [11, 35, 36], we found an association between hyperleptinemia and risk of metabolic syndrome independent of proinflammatory cytokines among women.

Several theories have been proposed to explain the mechanism for leptin resistance. The leptin stimulated SOCS-3 downregulates the JAKSTAT pathways and inhibits leptin signaling [37, 38]. Expression of SOCS-3 amplifies along with the increase in fat mass and abdominal adiposity during aging, as well as an increase in insulin resistance, suggesting that it could be a mediator of leptin resistance in aging individuals [4, 39, 40]. Thus, the relatively greater leptin insensitivity among women in our study may also be explained by elevated serum leptin levels, which may impair leptin signaling due to increased production of SOCS-3 resulting in leptin resistance. Leptin resistance in brain may lead to excess triglyceride accumulation in adipose tissue, liver, muscles, and pancreas, resulting in impaired insulin sensitivity and secretion [37].

There are several limitations of this study. Although there seem to be some differences in leptin levels by race in the present study, the sample size did not allow stratification beyond gender. However, all the analyses were adjusted for race. We used the outcome variable, metabolic syndrome, as it was defined using NCEP ATP II criteria in Health, Aging, and Body Composition study. The main difference between the new [41] and NCEP ATP III criteria is the inclusion of ethnicity specific cut-offs for waist circumference in the new criteria [41]. However, the new criteria do not have the waist circumference cut-off for African Americans, who are included in our study population. A previous study reported that the cut-offs were different among African American, European American, and Mexican American men but almost similar among women in the three ethnic groups [42]. The strength of this study is in its study design which allows the prospective examination between the serum leptin and the metabolic syndrome. The Health ABC study collected extensive information on life style and biochemical variables, which allowed for the adjustment of many potential confounders such as the use of hormone replacement therapy, number of hours fasted before blood draw, total calorie intake, and physical activity. Availability of detailed information on anthropometry including total body fat as measured by DEXA and abdominal visceral and abdominal subcutaneous fat as measured by CT scan allowed us to study the association of leptin with metabolic syndrome after controlling for body fat distribution.

In conclusion, the current study suggests that serum leptin is prospectively associated with the development of metabolic syndrome independent of body adiposity and proinflammatory markers among older women. Measurement of body fat mass requires expensive and invasive techniques, limiting its use in large population studies [2]. BMI has been shown to be a good indicator of percent body fat [2], but a natural decrease in height among older adults may limit its use in this population [43]. The prevalence of metabolic syndrome increases with aging and since hyperleptinemia predicts body fat [2] and is also linked to metabolic syndrome elevated serum leptin levels may become a useful biomarker for predicting the risk of metabolic syndrome at the population level among older women. Additionally, the positive association of leptin and metabolic syndrome among older women with normal BMI, in our study, suggests that older women with elevated serum leptin levels are at increased risk of metabolic syndrome even at normal weight. Therefore, serum leptin levels may be considered in conjunction with BMI for identifying obesity associated metabolic disorders.

## Figures and Tables

**Table 1:** Baseline characteristics of men by serum leptin quintile^1^.

	Serum leptin quintile				
	1	2	3	4	5
Men (*n* (%))	110 (19.9)	110 (19.9)	111 (20.1)	110 (19.9)	111 (20.1)
Mean serum leptin (ng/mL)^3^	2.14 (±0.05)	3.78 (±0.05)^2^	5.61 (±0.06)^2^	8.13 (±0.09)^2^	15.81 (±0.75)^2^
Demographic and behavioral variables					
Age (years)^3^	75.4 (±0.29)	75.1 (±0.25)	74.9 (±0.29)	75.1 (±0.28)	75.2 (±0.28)
Race (% white)	68	70	74	65	61
Alcohol use (% any consumption)	65	69	63	58	61
Education (% completed high school)	74	79	75	76	81
Smoking (lifetime pack-years)^3^	18.87 (±2.41)	19.1 (±2.44)	23.9 (±3.05)	20.9 (±3.15)	22.87 (±2.91)^2^
Physical activity (kcal/kg/week)^3^	10.2 (±1.87)	12.89 (±2.69)	8.08 (±1.48)	9.97 (±2.10)	8.55 (±1.43)
Dietary and anthropometric variables					
Total calorie intake (kcal)^4^	2197 (±90.4)	1966 (±79.4)	2055 (±75.7)	2014 (±78.2)	2124 (±96.1)
BMI (kg/m^2^)^3^	23.76 (±0.22)	25.16 (±0.20)^2^	26.15 (±0.25)^2^	27.44 (±0.27)^2^	29.11 (±0.38)^2^
Total body fat (%)^3^	24.4 (±0.26)	27.5 (±0.30)^2^	28.82 (±0.27)^2^	31.05 (±0.32)^2^	32.86 (±0.42)^2^
Abdominal circumference (cm)^3^	91.29 (±0.67)	95.26 (±0.59)	98.3 (±0.69)^2^	101.9 (±0.67)^2^	105.32 (±0.94)^2^
Abdominal visceral fat (cm^2^)^3^	103.2 (±3.9)	123.9 (±4.4)^2^	140.6 (±4.5)^2^	154.57 (±5.5)^2^	183.98 (±7.5)^2^
Abdominal subcutaneous fat (cm^2^)^3^	149 (±4.21)	190 (±4.1)^2^	213.61 (±5.4)^2^	248.8 (±6.7)^2^	276.91 (±7.9)^2^
Clinical and biochemical variables					
Diastolic blood pressure (mmHg)^3^	71.9 (±1.23)	71.8 (±1.07)	71.97 (±1.25)	74.2 (±1.09)	74.1 (±1.24)
Systolic blood pressure (mmHg)^3^	132 (±1.81)	133 (±2.19)	133.2 (±1.9)	135.8 (±2.03)	132.1 (±2.29)
Fasting glucose (mg/dL)^3^	95.4 (±2.63)	97 (±2.58)	99.02 (±2.5)	97.4 (±1.92)^2^	99.6 (±1.7)^2^
HDL cholesterol (mg/dL)^3^	52.2 (±1.24)	50.6 (±1.2)	48.3 (±1.1)	48.5 (±1.1)	50.2 (±0.98)
Triglycerides (mg/dL)^3^	104.5 (±4.1)	102.6 (±3.97)	115.3 (±4.3)	114.4 (±5.1)	116.2 (±4.2)
C-reactive protein (*µ*g/mL)^3^	1.74 (±0.23)	2.03 (±0.3)	1.94 (±0.23)	2.26 (±0.24)^2^	2.07 (±0.16)^2^
PAI-1 (ng/mL)^3^	17.93 (±1.55)	17.9 (±1.25)	24.6 (±1.8)	22.29 (±1.5)^2^	29.8 (±2.0)^2^
IL-6 (pg/mL)^3^	1.98 (±0.17)	2.08 (±0.14)	2.14 (±0.18)	2.15 (±0.15)^2^	2.01 (±0.12)
TNF-alpha (pg/mL)^3^	2.98 (±0.10)	3.05 (±0.12)	3.45 (±0.30)	3.37 (±0.13)	3.36 (±0.17)^2^

^1^Means (±SEM) are provided in the table unless specified.

^2^Significantly different from leptin quintile 1, *P* ≤ 0.05 (Dunnett's test for continuous variables and chi-square test for categorical variables).

^3^Values from baseline of the Health ABC study.

^4^Values from year 2 of the Health ABC study.

**Table 2:** Baseline characteristics of women by serum leptin quintile^1^.

	Serum leptin quintile				
	1	2	3	4	5
Women (*n* (%))	113 (19.9)	114 (20.7)	113 (19.9)	114 (20.7)	114 (20.7)
Mean serum leptin (ng/mL)^3^	5.24 (±0.21)	10.01 (±0.11)^2^	15.28 (±0.18)^2^	21.97 (±0.23)^2^	39.67 (±1.1)^2^
Demographic and behavioral factors					
Age (years)^3^	75.35 (±0.30)	75.35 (±0.30)	75.05 (±0.29)	74.76 (±0.27)	75.27 (±0.28)
Race (% white)	80	73	71	45^2^	41^2^
Alcohol use (% any consumption)	44	49	50	45	42
Education (% completed high school)	88	87	84	71^2^	78^ 2^
Smoking (lifetime pack-years)^3^	13.5 (±2.5)	8.29 (±1.70)	12.7 (±2.3)	11.3 (±2.27)	5.75 (±1.43)^2^
Physical activity (kcal/kg/week)^3^	5.52 (±1.04)	5.75 (±1.05)	5.72 (±1.24)	2.83 (±0.65)	7.31 (±2.76)
Dietary and anthropometric variables					
Total calorie intake (kcal)^4^	1612 (±63.6)	1793 (±85.4)	1650 (±68.0)	1690 (±67.3)	1654 (±64.9)
BMI (kg/m^2^)^3^	22.16 (±0.32)	24.17 (±0.31)^2^	25.66 (±0.34)^2^	27.9 (±0.41)^2^	30.3 (±0.46)^2^
Total body fat (%)^3^	34.18 (±0.52)	38 (±0.43)^2^	39.76 (±0.39)^2^	42.3 (±0.35)^2^	44.7 (±0.37)^2^
Abdominal circumference (cm)^3^	84.74 (±1.06)	88.9 (±0.98)	95.5 (±1.08)^2^	99.7 (±1.56)^2^	103.88 (±1.33)^2^
Abdominal visceral fat (cm^2^)^3^	78.6 (±3.8)	104.3 (±4.6)^2^	109.3 (±4.56)^2^	121 (±4.2)^2^	139.45 (±4.74)^2^
Abdominal subcutaneous fat (cm^2^)^3^	219.9 (±8.3)	267 (±7.8)^2^	306.2 (±9.1)^2^	370 (±9.5)^2^	418.12 (±11.3)^2^
Clinical and biochemical variables					
Diastolic blood pressure (mmHg)^3^	69 (±1.1)	71 (±1.13)	66.94 (±1.2)	71 (±1.09)	68.5 (±1.1)
Systolic blood pressure (mmHg)^3^	130 (±2.14)	137 (±2.01)^2^	131.1 (±1.9)	135 (±2.1)	132.2 (±2.19)
Fasting glucose (mg/dL)^3^	87 (±0.84)	91 (±1.6)	91.2 (±1.8)	92.8 (±1.1)^2^	92.0 (±0.78)^2^
HDL cholesterol (mg/dL)^ 3^	67 (±1.37)	68 (±1.7)	64.9 (±1.48)	63.7 (±1.49)	64.3 (±1.44)
Triglycerides (mg/dL)^3^	101.45 (±3.7)	113 (±4.99)	113 (±5.0)	110.4 (±4.20)	111.7 (±4.31)
C-reactive protein (*µ*g/dL)^3^	1.52 (±0.16)	2.33 (±0.31)	2.27 (±0.18)	3.18 (±0.33)^2^	3.3 (±0.56)^2^
PAI-1 (ng/mL)^3^	17.5 (±1.43)	24.3 (±2.57)	22.2 (±2.10)	28.9 (±2.10)^2^	27.5 (±2.18)^2^
IL-6 (pg/mL)^3^	1.78 (±0.16)	1.74 (±0.16)	1.96 (±0.21)	2.67 (±0.28)^2^	2.17 (±0.18)
TNF-alpha (pg/mL)^3^	2.76 (±0.11)	2.98 (±0.11)	2.94 (±0.11)	3.13 (±0.12)	3.31 (±0.25)^2^

^1^Means (±SEM), unless otherwise specified.

^2^Significantly different from leptin quintile 1, *P* ≤ 0.05 (Dunnett's test for continuous variables and chi-square test for categorical variables).

^3^Values from baseline of the Health ABC study.

^4^Values from year 2 of the Health ABC study.

**Table 3:** Adjusted odd ratios (OR) of the incidence of metabolic syndrome at year 6 by serum leptin quintile among men.

	Serum leptin quintile					
	1	2	3	4	5	*P* for trend^1^
Men (*n*)	110	110	111	110	111	
Serum leptin quintile (mean ± SEM)	2.14 (±0.05)	3.78 (±0.05)	5.61 (±0.06)	8.13 (±0.09)	15.81 (±0.75)	<0.0001
Incidence of metabolic syndrome at year 6 of study (*n*)	13	11	18	23	29	0.0076
Model 1^2^						
OR (95% CI)	1	0.83 (0.35–1.98)	1.36 (0.62–2.99)	1.88 (0.87–4.05)	2.69 (1.29–5.64)	0.0002
Model 2^3^						
OR (95% CI)	1	0.63 (0.25–1.58)	0.90 (0.38–2.14)	1.01 (0.40–2.54)	1.03 (0.37–2.86)	0.1435
Model 3^4^						
OR (95% CI)	1	0.65 (0.26–1.63)	0.73 (0.30–1.76)	0.77 (0.29–2.01)	0.68 (0.23–2.01)	0.5034

^1^Tests for linear trend used leptin as a continuous variable in logistic regression.

^2^Model 1: adjusted for age, race, site, years of education, alcohol use, smoking, and physical activity (testosterone level and numbers of hours fasted were not significant and did not affect the association between predictor and outcome, so they were not included in the model).

^3^Model 2: adjusted for variables in model 1 plus BMI (when BMI was replaced in the model with total percent fat, visceral fat, or subcutaneous fat the association between leptin and metabolic syndrome remained the same, so they were not included in the final analysis).

^4^Model 3: adjusted for variables in model 2 plus CRP and PAI-1 (TNF-alpha and IL-6 were not significant and did not affect the association between main predictor and the outcome, so they were not included in the final analysis).

**Table 4:** Adjusted odd ratios (OR) of the incidence of metabolic syndrome at year 6 by serum leptin quintile among women.

	Serum leptin quintile					
	1	2	3	4	5	*P* for trend^1^
Women (*n*)	113	114	113	114	114	
Serum leptin quintile (mean ± SEM)	5.24 (±0.21)	10.01 (±0.11)	15.28 (±0.18)	21.97 (±0.23)	39.67 (±1.1)	<0.0001
Incidence of metabolic syndrome at year 6 of study (*n*)	8	21	20	27	34	0.0003
Model 1^2^						
OR (95% CI)	1	3.29 (1.36−7.95)	3.25 (1.33−7.93)	5.21 (2.16−12.56)	7.97 (3.30−19.24)	<0.0001
Model 2^3^						
OR (95% CI)	1	3.04 (1.21−7.62)	2.94 (1.12−7.71)	4.56 (1.65−12.61)	6.38 (2.09−19.42)	0.0024
Model 3^4^						
OR (95% CI)	1	2.85 (1.12−7.22)	2.88 (1.091−7.59)	4.23 (1.53−11.7)	5.96 (1.95−18.13)	0.0098

^1^Tests for linear trend used leptin as a continuous variable in logistic regression.

^2^Model 1: adjusted for age, race, site, years of education, alcohol use, smoking, and physical activity (hormone replacement therapy and numbers of hours fasted were not significant and did not affect the association between predictor and outcome, so they were not included in the model).

^3^Model 2: adjusted for variables in model 1 plus BMI (when BMI was replaced in the model with total percent fat, visceral fat, or subcutaneous fat the association between leptin and metabolic syndrome remained the same, so they were not included in the final analysis).

^4^Model 3: adjusted for variables in model 2 plus CRP and PAI-1 (TNF-alpha and IL-6 were not significant and did not affect the association between main predictor and the outcome, so they were not included in the final analysis).

**Table 5:** Adjusted odd ratios (OR) of metabolic syndrome by BMI category.

	BMI ≤ 25 (normal)	BMI > 25 (high)
Men (*n*)	210	372
Mean serum leptin (ng/mL) (mean ± SD)	4.61 (±3.5)	8.44 (±6.3)
Metabolic syndrome cases (*n*)	18	91
OR (95% CI)	1.08 (0.49−2.40)	1.32 (0.90−1.94)
Women (*n*)	267	329
Mean serum leptin (ng/mL) (mean ± SD)	11.44 (±8.8)	24.50 (±13.6)
Metabolic syndrome cases (*n*)	37	91
OR (95% CI)^1,2^	2.10 (1.20−3.67)	2.00 (1.22−3.26)

^1^Adjusted for age, race, site, years of education, alcohol use, smoking, and physical activity.

^2^Odd ratios refer to the association of leptin with increased risk of metabolic syndrome among women with BMI ≤ 25 and those with BMI above 25.
